# The relative contribution of common and rare genetic variants to ADHD

**DOI:** 10.1038/tp.2015.5

**Published:** 2015-02-10

**Authors:** J Martin, M C O'Donovan, A Thapar, K Langley, N Williams

**Affiliations:** 1 MRC Centre for Neuropsychiatric Genetics and Genomics, Institute of Psychological Medicine and Clinical Neurosciences, Cardiff University School of Medicine, Cardiff, UK; 2 School of Psychology, College of Biomedical and Life Sciences, Cardiff University, Cardiff, UK

## Abstract

Attention deficit hyperactivity disorder (ADHD) is highly heritable. Genome-wide molecular studies show an increased burden of large, rare copy-number variants (CNVs) in children with ADHD compared with controls. Recent polygenic risk score analyses have also shown that *en masse* common variants are enriched in ADHD cases compared with population controls. The relationship between these common and rare variants has yet to be explored. In this study, we tested whether children with ADHD with (*N*=60) a large (>500 kb), rare (<1% frequency) CNV differ by polygenic risk scores for ADHD to children with ADHD without such CNVs (*N*=421). We also compared ADHD polygenic scores in ADHD children with and without CNVs with a group of population controls (*N*=4670; of whom *N*=397 had CNVs). The results show that children with ADHD with large, rare CNVs have lower polygenic scores than children without such CNVs (odds ratio (OR)=0.73, *P*=0.023). Although ADHD children without CNVs had higher scores than controls (OR=1.18, *P*=0.0031), this difference was not observed for ADHD children with CNVs (OR=0.86, *P*=0.27). These results are consistent with a polygenic liability threshold model of ADHD with both common and rare variants involved.

## Introduction

Attention deficit hyperactivity disorder (ADHD) is a childhood-onset neurodevelopmental disorder characterized by developmentally inappropriate levels of hyperactivity, impulsivity and inattention. Twin studies consistently indicate that it is highly heritable.^1^ Molecular genetic studies are beginning to suggest that the genetic architecture of ADHD is complex, with multiple common and rare genetic variants involved.^2, 3^ Recent molecular genetic studies using a method called ‘genomic-relationship-matrix restricted maximum likelihood' as implemented by the software Genome-wide Complex Trait Analysis confirm that additive common genetic variants contribute to estimates of heritability.^4, 5^ Although, genome-wide association studies (GWAS) thus far have not reported any significantly associated single-nucleotide polymorphisms (SNPs), these studies have been relatively small and underpowered for detecting individual loci significant at a genome-wide level,^2, 6^ compared with GWAS of adult psychiatric conditions such as schizophrenia.^7^ Other factors, including use of ‘pseudo-controls' in trio-based analyses^4^ and sample heterogeneity (in terms of phenotype, as well as ancestry), may have also had a role. However, an aggregate score (that is, a ‘polygenic risk' score) of common genetic variants was found to be, on average, higher in ADHD cases than in controls.^8^ For now, the power of this analysis is weak, though it is likely to improve with score alleles derived from future larger discovery GWAS samples, as has been seen in genetic studies of other psychiatric conditions, for example schizophrenia.^7^

In terms of rare variants, several studies have implicated an excess of large (>100 kb or >500 kb), rare (<1% population frequency) copy-number variants (CNVs) in ADHD cases compared with controls.^3, 5, 9^ However, only a small proportion of affected children have such large deletions or duplications and only a few specific loci (for example, duplications at 15q13.3 and 16p13.11) have been found to be significantly enriched in ADHD cases relative to controls.^3, 9^ Nevertheless, there is evidence that CNVs in children with ADHD are enriched in gene sets that are also enriched for common variant associations, suggesting that common and rare variants disrupt similar biological pathways in ADHD, such as those involved in cholesterol homeostasis or central nervous system development.^2^ Also, a pathway analysis of the top SNPs identified in ADHD GWAS has implicated a protein network related to neurite outgrowth, which also appears to be disrupted by several CNVs previously reported in ADHD cases.^10^

A question that has not yet been addressed is how common and rare genetic variants jointly contribute to individual liability. A polygenic liability threshold model of disorder implies that affected individuals with large, rare CNVs (which, in theory, might confer a more substantial amount of trait liability at the level of an individual carrier than can be seen at a population level) should, on average, have lower polygenic risk loading than affected individuals without such CNVs. To address this question, we analysed previously derived^8^ polygenic risk scores in a sample of children with ADHD for whom we also had CNV data. Risk scores were based on association data from an independent ADHD case–control GWAS.^6^ We hypothesized that children without a CNV would have a higher polygenic risk score for ADHD than those carrying a large and rare deletion or duplication.

## Materials and methods

Our cases were UK-based children with a research diagnosis of ADHD.^2^ ADHD was assessed using the Child and Adolescent Psychiatric Assessment,^11^ a semi-structured interview with parents, administered by trained research psychologists, under the weekly supervision of a child psychiatrist. Children with comorbid epilepsy, autism spectrum disorder (ASD), neurological or genetic disorders, Tourette's syndrome or bipolar disorder were excluded. Children with comorbid intellectual disability (ID), as defined by an IQ<70, were included in the sample (*N*=45). Ethical approval was obtained from the North West England and Wales Multicentre Research Ethics Committees and written informed consent/assent was obtained from parents and children. Controls were provided by the Wellcome Trust Case Control Consortium-Phase 2, consisting of 3000 individuals born in the UK during 1 week in 1958 (that is, the 1958 British Birth Cohort) and 3000 individuals from the UK Blood Services collection.^12^ Controls were unscreened for ADHD or other psychiatric problems and were not matched with the cases on the basis of any phenotypes as such data were not available.

Quality control of SNP data has been described previously.^2^ Analyses were based on the 502 542 SNPs, which passed quality control. The polygenic score and CNV data have been used for previous analyses in this sample; as such, the method is presented in brief, with further information on quality control, CNV calling procedures and polygenic score generation available from the previously published work.^2, 3, 8^ Polygenic scores were calculated in this sample using the Psychiatric Genomics Consortium ADHD GWAS meta-analysis (consisting of 2064 trios, 896 cases and 2455 controls) as the discovery sample.^6^ The polygenic scores for our primary analysis were calculated in PLINK^13^ for LD-pruned SNPs reaching a *P*-value threshold of <0.5 in the main discovery sample, as previously published.^8^ Polygenic scores were additionally calculated at several other thresholds (0.4, 0.3, 0.2, 0.1 and 0.05) using the same method.

As reported previously,^2, 3^ CNVs >500 kb were called in the sample using PennCNV and limited to those CNVs which had a population (cases and controls) frequency of <1%. Primary analyses were limited to these CNVs because they have better concordance across different genotyping platforms, and are determined with greater accuracy and are more robustly associated with neurodevelopmental disorders.^3^ Data on CNVs and polygenic scores were available for *N*=481 children with ADHD, aged 6–17 years (mean=10.9, s.d.=2.8), of whom *N*=67 (13.9%) were female. Control samples consisted of *N*=5081 individuals with SNP data, of whom *N*=4670 (*N*=2375 males and *N*=2295 females) had both polygenic score and CNV data available. Of note, 12.5% (*N*=60) of the children with ADHD and 8.5% (*N*=397) of the controls had a rare CNV>500 kb.

The polygenic scores were based on a set of *N*=46 156 risk alleles (at threshold *P*<0.5) and were standardized using *z*-score transformations. A logistic regression was used to determine whether polygenic risk scores were higher in children with ADHD with CNVs than in those without. Following on from previous work,^8^ logistic regressions were also used to test whether higher polygenic scores predicted case or control status, separately for children with ADHD with and without CNVs. In line with previous analyses, two principal components (calculated using the EIGENSTRAT software package and found to have maximum impact on the genomic control inflation factor in the original GWAS analysis) were entered into all the regression analyses as covariates to control for population stratification.^2, 8^ All analyses were also co-varied for gender. For each association test, the amount of variance explained was calculated as the difference of Nagelkerke pseudo-*R*^2^ in the full regression model (including all covariates and polygenic risk score), as compared with the reduced model (containing only the population covariates and gender). All analyses were performed in Stata version 13.1.

### Secondary analyses

The main analyses (comparing children with ADHD with and without CNVs) were re-run using polygenic scores based on the different thresholds (0.4, 0.3, 0.2, 0.1 and 0.05). The number of SNPs in each of these sets was: 37 078, 28 005, 18 723, 9519 and 4845, respectively. The analyses were also re-run expanding the list of CNVs in cases to any that were >200 kb (*N* children with=139; *N* children without=342). Additive and multiplicative models of CNV presence and polygenic risk scores were also tested.

## Results

**Figure 1:**
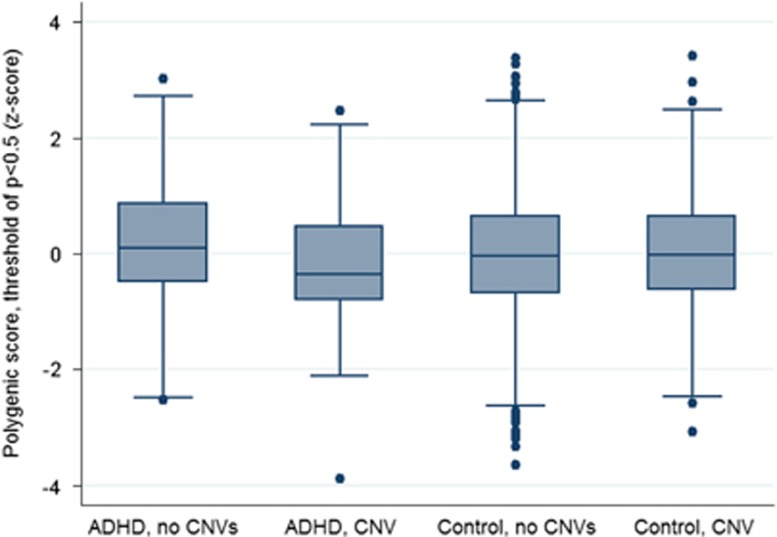
Box plot of mean standardized polygenic score in ADHD cases and controls, with and without CNVs>500 kb. ADHD, attention deficit hyperactivity disorder; CNV, copy-number variant.

Children with a CNV>500 kb had a significantly lower polygenic risk score than those without such a CNV (odds ratio (OR)=0.73, 95% confidence intervals (CI)=0.55–0.96, *P*=0.023, *R*^2^=0.015). A two-tailed permutation test of CNV status in the case group (*N*=481) using 10 000 replicates supported these results (*P*=0.0224, SE(*P*)=0.0015). Figure 1 displays box plots of the polygenic scores of children with ADHD and controls, split by those with and without a CNV>500 kb. Although we have previously shown that children with ADHD have higher polygenic risk scores for ADHD than population controls,^8^ limiting the analysis to children with CNVs (*N*=60) shows no difference between cases and controls (OR=0.86, 95% CI=0.67–1.12, *P*=0.27, *R*^2^=0.002), although this may be due to the small sample size of children with ADHD with large, rare CNVs. On the other hand, restricting the analysis to children with ADHD without large CNVs (*N*=421) confirms that these children have higher polygenic scores as compared with controls (OR=1.18, 95% CI=1.06–1.31, *P*=0.0031, *R*^2^=0.003).

**Figure 2:**
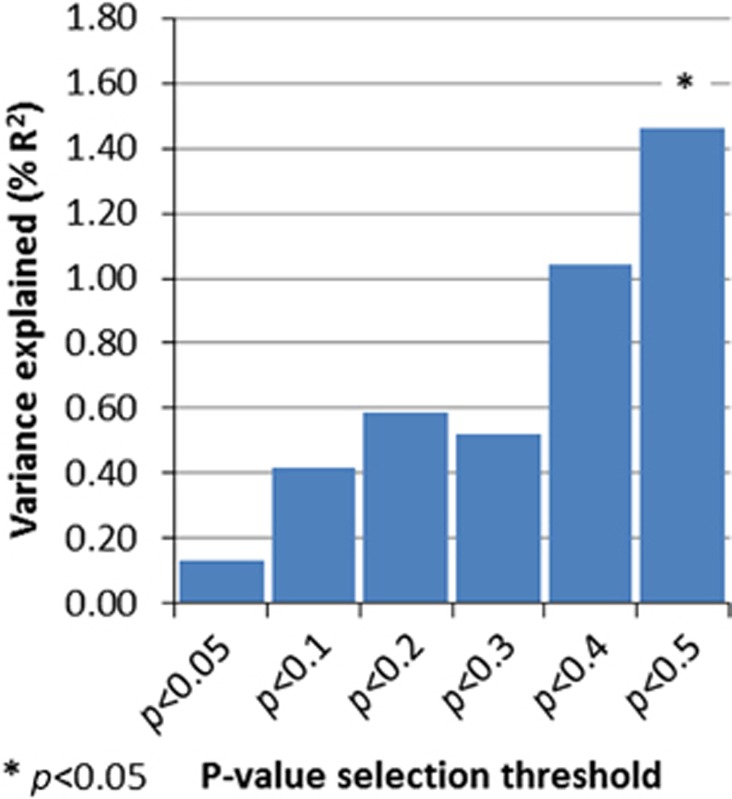
Variance explained (*R*^2^) in analysis comparing ADHD children with and without CNVs>500 kb, using polygenic scores calculated at a variety of different thresholds. ADHD, attention deficit hyperactivity disorder; CNV, copy-number variant.

**Figure 3:**
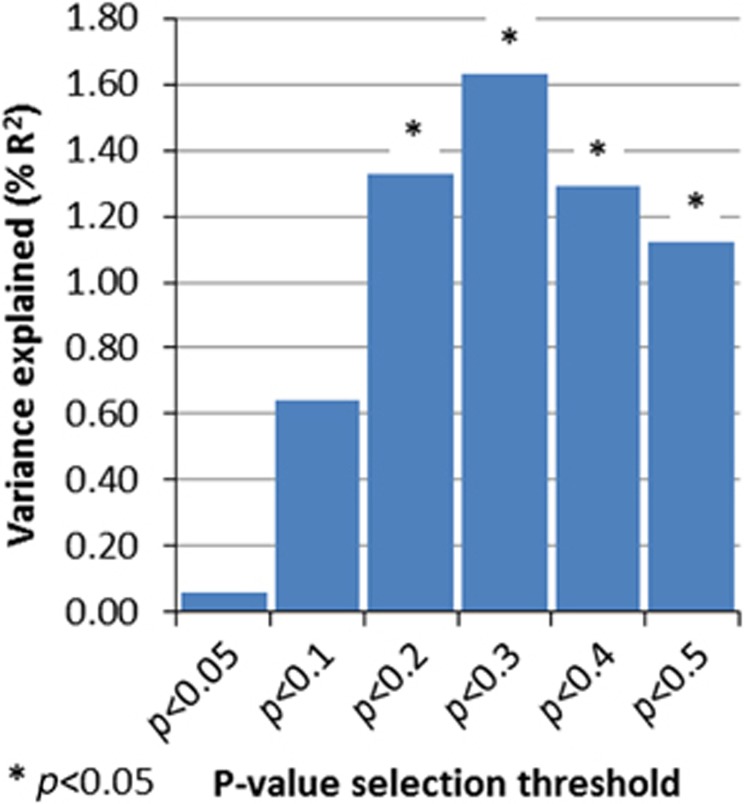
Variance explained (*R*^2^) in analysis comparing ADHD children with and without CNVs>200 kb, using polygenic scores calculated at a variety of different thresholds. ADHD, attention deficit hyperactivity disorder; CNV, copy-number variant.

Secondary analyses using different *P*-value thresholds for calculating polygenic scores demonstrated that significant effects are seen only at the least stringent *P*-value threshold (*P*<0.5), a finding that is consistent with other work based on polygenic score analysis when risk alleles are derived from modest sample sizes.^14^ See Figure 2 for the results using all the thresholds. Analyses using the primary threshold for selecting risk alleles (*P*<0.5) and including smaller CNVs>200 kb also showed higher polygenic scores in those without CNVs (OR=0.77, 95% CI=0.63–0.95, *P*=0.012, *R*^2^=0.011). Significant results were additionally attained at more of the risk allele thresholds (see Figure 3). Excluding children with ID (*N*=45, of whom *N*=10 had a large, rare CNV) did not alter the main results (OR=0.66, 95% CI=0.49–0.90, *P*=0.0086, *R*^2^=0.023).

To test an additive model including both common and rare variants, a multivariate logistic regression was run with both ADHD polygenic risk scores and CNV presence as predictors, all the covariates, and with case status as the outcome. Both polygenic scores (OR=1.15, 95% CI=1.04–1.27, *P*=0.009, *R*^2^=0.002) and CNV presence (OR=1.63, 95% CI=1.17–2.26, *P*=0.004, *R*^2^=0.003) showed independent associations with case status. A multiplicative model adding an interaction term of ‘CNV presence × polygenic risk score' showed a significant interaction term (OR=0.71, 95% CI=0.51–0.98, *P*=0.036, *R*^2^=0.001).

## Discussion

These results imply that children with ADHD with a large, rare CNV (>500 kb or >200 kb) require less of a polygenic burden of common alleles to manifest the disorder. The polygenic risk score loading was relatively low in ADHD children with large, rare CNVs. However, the difference in polygenic loading between them and controls was not significant, although this may have resulted from low power. The results of this study are consistent with a polygenic liability threshold model of ADHD, with both common SNPs and rare CNVs involved in ADHD disease risk. Additive and multiplicative models support these results. Both CNV presence and higher polygenic risk scores were found to be independently associated with case status. Furthermore, the significant interaction term in the multiplicative model supports the main result of this study in that those with a CNV and lower polygenic score are more likely to be cases.

Although these results could be interpreted to suggest that there may be common and rare variant forms of ADHD, it is important to bear in mind that the effect sizes of this and previous studies are quite small and it is not yet clear whether these are truly distinct variants of the disorder. We have previously shown that children with ADHD with a large, rare CNV do not differ clinically (beyond a greater likelihood of ID) or in terms of family history of psychiatric disorders or parental ADHD symptoms from children with ADHD without such CNVs.^15^ This suggests that there may be no clear and distinct forms of ADHD but further work examining this issue is needed. Interestingly, CNVs found in children with ADHD appear to disrupt similar biological processes to those hit by top GWAS SNPs,^2, 10^ which suggests that even if there are different aetiological routes to ADHD, they may act on similar biological mechanisms.

Given the known pleiotropy of genetic risks for various neurodevelopmental, psychiatric and neurological outcomes,^3, 4, 16, 17^ it is possible that some individuals with both a pathogenic CNV and a greater than average polygenic loading were not recruited to the ADHD sample by virtue of having another primary neurodevelopmental disorder, which may share aetiology with ADHD (for example, ASD, severe ID or epilepsy) and would have meant that they did not meet study inclusion criteria. These inclusion criteria are typical of ADHD studies and are intended to increase sample homogeneity. The recent publication of the DSM-5 saw the removal of previous diagnostic exclusions relevant to ADHD (for example, an ASD diagnosis excluding a diagnosis of ADHD in DSM-IV). As use of the DSM-5 becomes more widespread, research samples for genetic analyses of childhood disorders, such as ADHD may become more clinically representative. Although studies do find an increased burden of potentially pathogenic CNVs in children with ID and ASD^18^ and another study has reported an increased polygenic loading of ASD common variants in an independent sample of children with ASD,^19^ it is not yet known whether children with more complex neurodevelopmental problems (for example, those with multiple comorbidities) are more likely to have both types of risk variants (common and rare) than children with ADHD without these comorbidities.

Children with CNVs and high polygenic scores may have been less likely to be ascertained for other reasons. For example, those who are most severely affected may have not agreed to participate, whereas those families in which the genetic loading is highest may also have more chaotic family structures, which is likely to be a barrier for study participation.

Future ADHD studies should aim to determine whether the pattern of results observed in the current study is driven by particular CNV risk loci confirmed to be relevant to ADHD. Current ADHD sample sizes are too small to implicate many specific CNV loci in the disorder.^3, 9^ Similarly, as ADHD GWAS sample sizes increase, so will the power to robustly detect associated common variants. The current analyses are limited by existing small sample sizes and would therefore benefit from replication in larger future samples.

Like other psychiatric conditions (for example, ASD or schizophrenia), ADHD is an aetiologically complex and highly heritable condition. The results of the present study provide the first empirical molecular genetic support for the hypothesis that risk for ADHD follows a polygenic liability threshold model, in which individuals with large, rare CNVs require less loading of multiple common genetic risk variants for developing ADHD. A similar pattern, where the liability threshold for disorder is shifted downwards, might also be seen for other types of rare genetic mutations and will require future investigation.
